# Fatty acids and oxidative stress in psychiatric disorders

**DOI:** 10.1186/1471-244X-8-S1-S5

**Published:** 2008-04-17

**Authors:** Sofia Tsaluchidu, Massimo Cocchi, Lucio Tonello, Basant K Puri

**Affiliations:** 1 Università di Bologna, Bologna, Italy; 2 MRI Unit, MRC Clinical Sciences Centre, Imaging Sciences Department, Imperial College London, Hammersmith Hospital, Du Cane Road, London W12 0HS, UK

## Abstract

**Background:**

The aim of this study was to determine whether there is published evidence for increased oxidative stress in neuropsychiatric disorders.

**Methods:**

A PubMed search was carried out using the MeSH search term 'oxidative stress' in conjunction with each of the DSM-IV-TR diagnostic categories of the American Psychiatric Association in order to identify potential studies.

**Results:**

There was published evidence of increased oxidative stress in the following DSM-IV-TR diagnostic categories: mental retardation; autistic disorder; Rett's disorder; attention-deficit hyperactivity disorder; delirium; dementia; amnestic disorders; alcohol-related disorders; amphetamine (or amphetamine-like)-related disorders; hallucinogen-related disorders; nicotine-related disorders; opioid-related disorders; schizophrenia and other psychotic disorders; mood disorders; anxiety disorders; sexual dysfunctions; eating disorders; and sleep disorders.

**Conclusion:**

Most psychiatric disorders are associated with increased oxidative stress. Patients suffering from that subgroup of these psychiatric disorders in which there is increased lipid peroxidation might therefore benefit from fatty acid supplementation (preferably with the inclusion of an antioxidant-rich diet) while patients suffering from all these psychiatric disorders might benefit from a change to a whole-food plant-based diet devoid of refined carbohydrate products.

## Background

Fatty acids are an important constituent of cellular membranes. The membranes are able to synthesize them with acetyl-CoA but are not capable of synthesizing essential fatty acids (linoleic acid and alpha-linoleic acid), which are the precursors to, respectively, arichidonic acid (an omega-6 long-chain polyunsaturated fatty acid) and docosahexaenoic acid (an omega-3 long-chain polyunsaturated fatty acid). The cellular membrane, with its high content of unsaturated fatty acids, plays a protective, anti-inflammatory role and indirectly an antioxidant role, favouring physiological defence processes against free radicals [1,2].

Oxidative stress is a condition which modifies the normal intracellular balance between oxidant substances produced during aerobic metabolism and antioxidant system processes which perform the function of neutralization, putting a series of protective mechanisms, of both an enzymatic and non-enzymatic nature, in action. Enzymatic systems include superoxide dismutase (SOD), catalase (CAT) and glutathione peroxidase (GSH-Px). In non-enzymatic systems, the most important molecules are glutathione, alpha-tocopherol (vitamin E), ascorbic acid (vitamin C), flavonoids, the phenol compounds and the minerals zinc (Zn), copper (Cu) and selenium (Se) [3].

Numerous physiological and pathological processes such as ageing, excessive caloric intake, infections, inflammatory disorders, environmental toxins, pharmacological treatments, emotional or psychological stress, ionizing radiation, cigarette smoke and alcohol increase the bodily concentration of oxidizing substances, known as reactive oxygen species (ROS) or, more commonly, free radicals. These are chemical species which are highly reactive owing to the presence of free unpaired electrons. An increase in free radicals compromises the delicate homeostatic mechanisms which involve neurotransmitters, hormones, oxidizing substances and numerous other mediators.

Owing to their structure, which is rich in double bonds, polyunsaturated fatty acids (PUFAs) render cellular membranes vulnerable to damage from free radicals, causing peroxidation. The damage induced by lipid peroxidation renders the cell unstable, and therefore compromises fluidity, permeability, signal transduction and causes receptor, mitochondrial DNA and nuclear alterations.

Oxidizing stress from free radicals is one of the factors which contributes to an increase in the speed of the cell cycle and consequent premature cell death, leading to many degenerative illnesses in the central nervous system, as well as psychiatric disturbances. Peripheral systems undergo a process of atherogenesis and can lead to pathologies in the cardiovascular system.

The aim of this study was to determine whether there is published evidence for increased oxidative stress in neuropsychiatric disorders.

## Methods

A PubMed search was carried out at the web site , using the MeSH search term 'oxidative stress' in conjunction with each of the DSM-IV-TR diagnostic categories of the American Psychiatric Association. The abstract, and, if necessary, the full paper, of any positive 'hit' was then perused further to determine whether or not the hit was valid.

## Results

There was published evidence of increased oxidative stress in the following DSM-IV-TR diagnostic categories: mental retardation; autistic disorder; Rett's disorder; attention-deficit hyperactivity disorder; delirium; dementia; amnestic disorders; alcohol-related disorders; amphetamine (or amphetamine-like)-related disorders; hallucinogen-related disorders; nicotine-related disorders; opioid-related disorders; schizophrenia and other psychotic disorders; mood disorders; anxiety disorders; sexual dysfunctions; eating disorders; and sleep disorders.

## Discussion

Our study indicates that there is an association between increased oxidative stress and the majority of psychiatric disorders. These data are important for recognizing that the integrity and functionality of biomolecules is closely correlated with integration with fatty acids and antioxidants, whether from a dietary-habit point of view or from a therapeutic point of view.

The long hydrocarbon chain of polyunsaturated fatty acids in membrane phospholipids has kinks at the site of *cis *double bonds which, as a result of the steric effects created, provides remarkable reactivity to the molecule. Owing to the fluidity of the lipid component, the biomolecules demonstrate a notable degree of mobility, thereby helping to explain their functional properties.

As mentioned earlier, neuronal membrane phospholipid PUFAs are particularly susceptible to peroxidation mediated by oxyradicals, that is, by ROS. These constitute a highly reactive species, owing to the presence of unpaired outer shell electrons, and are generated both through aerobic metabolic physiological processes and through pathological processes which may be ischaemic, inflammatory, or caused by emotional or psychological stress or environmental pollution, smoking or poor dietary habits.

In membrane lipid peroxidation, the damage to lipids occurs in three stages [3]. The first stage, initiation, involves the attack of a reactive oxygen metabolite capable of abstracting a hydrogen atom from a methylene group in the lipid. The presence of a double bond adjacent the methylene group weakens the bond between the hydrogen and carbon atoms so that it can easily be removed from the molecule. Following hydrogen abstraction, the remaining fatty acid radical R^• ^retains one electron and is stabilized by rearrangement of the molecular structure to form a conjugated diene. When oxygen is present at a high enough concentration in the surrounding environment, the fatty acid radical will react with it to form a lipid peroxy radical (ROO^•^) during the propagation stage. The ROO^• ^is capable of abstracting another hydrogen atom from a neighboring fatty acid, which undergoes the same reaction rearrangement and interaction with oxygen. The ROO^• ^becomes a lipid hydroperoxide that, in the presence of transitional ions, can further decompose to an aldehyde or form cyclic endoperoxide, isoprostanes and hydrocarbons. The propagation stage allows the reaction to continue. A single initiation can lead to a chain reaction resulting in peroxidation of the entire unsaturated lipid in the membrane. Particular antioxidants, such as vitamin E, can stop this process, and therefore are defined as chain-breaking antioxidants. Fatty acids with no double bonds or with just one double bond can undergo oxidation but not a chain lipid-peroxidation process; for example, oleic acid with 18 carbon atoms and one double bond (C18:1) does not undergo this lipid peroxidation process. The last stage, chain termination, occurs following interaction of one ROO^• ^with another radical or antioxidant [3,4].

Thus the mechanism of action of the oxidative stress might, in some cases, be related to increased membrane lipid peroxidation. For example, it has recently been found that in schizophrenia there is an increase in the levels of ethane, which is a direct marker of *n*-3 lipid peroxidation [5].

There are two major potential therapeutic implications of these results. First, increased oxidative stress, associated with increased lipid peroxidation, reinforces the possibility that dietary supplementation with fatty acids may be helpful. There is already evidence that this may be the case in respect of depression, Huntington's disease and attention-deficit hyperactivity disorder [6,7].

Second, these results suggest that there may be merit in a change in diet to one which is rich in free radical scavengers. Such scavengers are primarily botanical in nature. There is already strong epidemiological, rodent and human evidence that a change in nutrition to one which is primarily a whole-food plant-based diet devoid of refined carbohydrate products (such as white refined sugar, white flour, white pasta and white polished rice) may help prevent and possibly even reverse important cardiovascular causes of morbidity and mortality of the western world, and may possibly even do so in respect of certain cancers [8]. If increased oxidative stress is an important component of most psychiatric disorders, then there can be no harm in recommending a change in diet to one which is more whole-food and plant-based; at the very least such patients should benefit in terms of lower blood pressure, a lower body mass index, reduced risk of cardiovascular disease and possibly reduced risk of diabetes mellitus and certain types of cancer.

## Conclusion

Most psychiatric disorders are associated with increased oxidative stress. Patients suffering from that subgroup of these psychiatric disorders in which there is increased lipid peroxidation might therefore benefit from fatty acid supplementation, while patients suffering from all these psychiatric disorders might benefit from a change to a whole-food plant-based diet.

## Competing interests

The author(s) declare that they have no competing interests.

## Authors' contributions

All the authors made substantial contributions to the design and conception of the study. BKP was particularly involved in data collection. All authors were involved in the interpretation of the data. All the authors have been involved in drafting and revising the manuscript and have read and approved the final manuscript.
